# State Anhedonia in Young Healthy Adults: Psychometric Properties of the German Dimensional Anhedonia Rating Scale (DARS) and Effects of the COVID-19 Pandemic

**DOI:** 10.3389/fpsyg.2021.682824

**Published:** 2021-06-23

**Authors:** Sarah A. Wellan, Anna Daniels, Henrik Walter

**Affiliations:** ^1^Research Division of Mind and Brain, Department of Psychiatry and Psychotherapy Campus Charité Mitte, Charité – Universitätsmedizin Berlin, corporate member of Freie Universität Berlin and Humboldt-Universität zu Berlin, Berlin, Germany; ^2^Berlin School of Mind and Brain, Faculty of Philosophy, Humboldt-Universität zu Berlin, Berlin, Germany

**Keywords:** anhedonia, pleasure, COVID-19, depression, mental health, confirmatory factor analysis, validity, reliability

## Abstract

Healthy reward processing is a complex interplay of several components. Recent self-report measures of anhedonia, the decrease or loss of hedonic capacity, take this complexity into account. The Dimensional Anhedonia Rating Scale (DARS) measures interest, motivation, effort and consummatory pleasure across four domains: hobbies, food/drink, social activities and sensory experiences. In the present cross-sectional survey study, we validated the German version of the DARS in a sample of 557 young healthy adults. Factor structure as well as convergent and divergent validity were assessed. As a secondary aim, we examined the effects of the COVID-19 pandemic on state anhedonia and depression severity. Our results suggest good convergent and divergent validity and high internal consistency of the German DARS. The original differentiation of four factors mapping onto the four domains was confirmed and measurement invariance before and during the COVID-19 pandemic was established. We conclude that the DARS is a valid instrument to comprehensively assess state anhedonia in healthy German samples. Future studies should further assess the utility of the German DARS in clinical contexts. In line with many previous studies, participants during the pandemic reported significantly higher levels of depressive symptoms compared to participants in the months before. We found no indication that the COVID-19 pandemic affected state hedonic capacity.

## Introduction

The ability to experience pleasure is an important part of subjective well-being and mental health (Kringelbach and Berridge, 2017). Preclinical research on reward processing has dissociated several subcomponents, with partially distinct neural correlates, interacting in a normal hedonic response (Berridge and Robinson, 2003). Hedonic capacity, therefore, not only involves the ability to experience pleasure at the receipt of a reward, i.e., consummatory pleasure, but also comprises reward anticipation, reward learning and decisional components, weighing the estimated efforts of pursuit against the subjective value of a reward (Treadway and Zald, 2011; Der-Avakian and Markou, 2012; Kringelbach et al., 2012; Rømer Thomsen, 2015; Husain and Roiser, 2018). Although the exact conceptualization of these reward components may vary in the literature, the important insight with respect to clinical research is that an impairment of any of these components could potentially lead to a severe reduction or loss of pleasure in one's life. Research on anhedonia, the decrease or loss of hedonic capacity, needs to take this complexity into account.

Anhedonia is a transdiagnostic symptom occurring, as state or trait, in several psychiatric and neurological disorders, including major depressive disorder (MDD; Treadway and Zald, 2011; Cooper et al., 2018), schizophrenia (Gard et al., 2007; Lambert et al., 2018), substance use disorder (Garfield et al., 2014), post-traumatic stress disorder (Nawijn et al., 2015) and Parkinson's disease (Assogna et al., 2011). In addition, anhedonia can be found in the general population (Chan et al., 2012; Dodell-Feder and Germine, 2018; Martino et al., 2018) and has been recognized as a risk factor for several mental disorders (Kwapil, 1998; Gooding et al., 2005; Keedwell et al., 2012; Stringaris et al., 2015; Luby et al., 2018; Guffanti et al., 2019; Ward et al., 2019). Identifying the exact hedonic dysfunction underlying the symptom of anhedonia and potentially distinguishing anhedonic subtypes are important goals of clinical research. For this purpose, measures of anhedonia on different units of analysis, e.g., on neurobiological, behavioral and self-report levels, have to be integrated (Cuthbert and Insel, 2013). This necessitates to be mindful of the exact reward processing components the applied measurement instrument targets. Here, we focused on measures of self-report, specifically the validation of a recent scale designed to capture different facets of state anhedonia: the Dimensional Anhedonia Rating Scale (DARS; Rizvi et al., 2015). The DARS is one of several newly developed anhedonia scales aiming to integrate findings from the neurobiology of reward processing while providing culturally unbiased and contemporary item content (Rizvi et al., 2016).

As a trait, anhedonia has traditionally been assessed with the Revised Chapman Physical Anhedonia Scale (CPAS) and the Revised Chapman Social Anhedonia Scale (CSAS; Chapman et al., 1976, 1980; Bailer et al., 2004). Both were developed as part of a larger set of scales specifically tailored to examine psychosis proneness. Their items comprise different hedonic components, such as anticipation, interest, consummation and effort, but also trait aspects that are not directly related to anhedonia. Since their length (61/40 items) is an impediment for clinical use and the item content has been criticized as culturally biased, new trait measures of anhedonia have been developed (Gard et al., 2006; Gooding and Pflum, 2014; Rizvi et al., 2016). The Temporal Experience of Pleasure Scale (TEPS; Gard et al., 2006) measures predominantly physical pleasure on an anticipatory and a consummatory subscale, whereas the Anticipatory and Consummatory Interpersonal Pleasure Scale (ACIPS; Gooding and Pflum, 2014) focuses on the social domain. For the ACIPS, factor analysis did not distinguish between anticipation and consummation but revealed three interpersonal subdomains instead (Gooding and Pflum, 2014).

As a newly developed state anhedonia scale, the DARS is most importantly compared to the Snaith-Hamilton Pleasure Scale (SHAPS; Snaith et al., 1995) which also measures state anhedonia and has been applied widely, in particular with regard to state anhedonia in depression (Rizvi et al., 2016; Trøstheim et al., 2020). The SHAPS measures hedonic tone during the last few days with 14 hypothetically formulated items, e.g., “I would be able to enjoy my favorite meal.” The item content exclusively targets consummatory pleasure but covers four domains: interests/pastimes, social interaction, sensory experience and food/drink. To avoid cultural, gender and age biases and enable widespread use the items are kept simple and, in part, very general (Snaith et al., 1995). A recent meta-analysis (Trøstheim et al., 2020) based on SHAPS scores of 246 adult samples reported significantly higher, i.e., more anhedonic, scores in patients compared to healthy adults. Patients were diagnosed with current MDD, bipolar disorder, schizophrenia, substance use disorder, Parkinson's disease or chronic pain. MDD patients scored higher than any other patient group, thereby indicating that anhedonia in MDD affects multiple domains of pleasure (Trøstheim et al., 2020).

In contrast to the SHAPS, the DARS (Rizvi et al., 2015) was designed to capture not only consummatory pleasure, but also interest/desire, motivation and effort across the same four domains, thereby offering an important addition to anhedonia research. The DARS comprises 17 items assessing state anhedonia right now. It avoids cultural, gender and age biases as well as too specific item content by asking the participants to provide two or three of their own favorite examples for each of the four domains. Principal component analysis revealed four components distinguishing between the four domains pastimes/hobbies, food/drink, social activities and sensory experiences. Hence, domain-specific subscales have been proposed and high internal consistency has been reported for all scales (Rizvi et al., 2015; Arrua-Duarte et al., 2019). So far, the DARS has demonstrated adequate convergent and divergent validity in community and clinical samples regarding the SHAPS, measures of behavioral inhibition and activation and depression severity (Rizvi et al., 2015; Arrua-Duarte et al., 2019). Sullivan-Toole et al. (2019) found correlations between the DARS and reward-related and effort-related parameter estimates in a novel effort-based decision-making task. In addition, the DARS has shown predictive value above the SHAPS regarding reward functioning and differentiation of treatment-resistant MDD subgroups (Rizvi et al., 2015). A Spanish validation study corroborated the four-factor structure in a diverse clinical sample (Arrua-Duarte et al., 2019).

The primary aim of the present study was to validate the German version of the DARS in a convenience sample of 557 young, mentally healthy adults. This, to the best of our knowledge, is the first report on the factor structure and psychometric properties of the German DARS in its original 17-item version. We expected confirmatory factor analysis (CFA) to corroborate the four-factor structure mapping onto the four domains. Since the differentiation of anhedonic components is an important question in anhedonia research, we additionally evaluated CFA models distinguishing interest, motivation, effort and consummatory pleasure. Based on the literature, we predicted an inferior fit for these models. As measures of convergent validity, we expected moderate correlations to SHAPS, TEPS, ACIPS, CPAS, CSAS, trait positive affect and behavioral activation sensitivity. We expected either no or only very low correlations to the diverging constructs of behavioral inhibition sensitivity and trait negative affect. Since only few items in depression scales specifically target anhedonia, we hypothesized to find low to moderate correlations to depression severity.

As a secondary aim, we assessed the impact of the coronavirus disease 2019 (COVID-19) pandemic on state anhedonia and depression severity. This was not part of the original aim of our study. However, since our data was collected from August 2019 to July 2020, a time during which the COVID-19 pandemic emerged and spread around the world, we added this secondary aim to our analysis and divided our sample into a pre-pandemic and a during-pandemic group. In line with many reports of an increase in depression rates and a deterioration of mental health (Henssler et al., 2020; O'Connor et al., 2020; Pappa et al., 2020; Bueno-Notivol et al., 2021; Cénat et al., 2021), we assumed to find higher depression scores in the during-pandemic group. Further, we examined differences in state anhedonia between both groups, as there is little research on the effects of the COVID-19 pandemic on state hedonic capacity yet (Moccia et al., 2021; Prati and Mancini, 2021). Since anhedonia is a core symptom of depression, it is well-possible that anhedonia levels increase along with depression scores (Moccia et al., 2021). There are reports on fewer positive experiences during lockdown (Klaiber et al., 2020). However, a recent meta-analysis showed no significant effect on positive psychological functioning (Prati and Mancini, 2021). Losing the opportunity to experience many of the usual leisure activities certainly is a consequence of COVID-19 mitigation strategies such as lockdowns (Mutz and Gerke, 2020). But losing the opportunity to do something you usually enjoy does not necessarily imply an impairment of the capacity to enjoy. Given the lack of research on the topic of anhedonia specifically during the COVID-19 pandemic, we conducted two-tailed tests to examine this question.

Adding this secondary aim to our analysis also contributed to the DARS validation, the focus of our study. Assessing state anhedonia with both SHAPS and DARS allowed us to examine potential differences between these measures which might result from their different set-ups. Moreover, dividing our sample into two groups enabled us to perform multigroup CFA on the DARS. We examined whether the COVID-19 pandemic had influenced the factor model of the DARS by testing measurement invariance across both groups and performed latent mean comparisons on all factors of the DARS. This, to the best of our knowledge, is the first report on measurement invariance of the DARS across severe changes to the social environment.

## Materials and Methods

### Procedure

Data collection took place during August 2019 and July 2020, as part of the ELAN [Enjoying Life – The (AN)hedonic Spectrum] study. Our online survey was created on the Unipark platform (www.unipark.com). The link was distributed via mailing lists, websites and social media, predominantly in Berlin and surrounding areas. After a detailed study description, participants gave their informed consent online. Participants were not compensated but were offered the opportunity to partake in a raffle of five €30 vouchers.

We only included participants aged 18 to 30 who did not report any previous or current psychiatric diagnosis. Our survey was applied in German, making German proficiency a prerequisite. *N* = 922 participants gave consent and started the survey. Participants who reported an age outside the range of 18 to 30 (*n* = 49), who answered yes to the question whether they had a past or present psychiatric or neurological illness (*n* = 87) or who answered yes to ever having used psychopharmacological medication (*n* = 59) were immediately led to an alternative end page. For the present analysis, we further excluded participants who aborted the survey (*n* = 302), failed to answer two control questions (e.g., “Please tick option 3 in this line”) correctly (*n* = 21 among those who completed the survey) or had missing values for the DARS (*n* = 3 among those who completed the survey). Missing DARS values entailed missing ratings and/or missing examples. Where multiple participations of the same person were identified, we excluded all participations after the first (*n* = 23 among those who completed the survey). This resulted in a final sample of *n* = 557. Of the *n* = 365 excluded participants, only *n* = 103 had any data on either the DARS, the SHAPS or the depression scale, i.e., the scales necessary for the analyses reported in this paper. These *n* = 103 excluded participants did not significantly differ from the *n* = 557 included participants regarding age, gender, education level and occupational status. Note that the description of our final sample as mentally healthy only refers to the participants' self-reports of having a mental disorder or taking psychopharmacological medication, presently or in the past. This does not preclude clinically relevant mental health symptoms that might not have been diagnosed or recognized yet. Although we asked for other physical illnesses, we did not exclude participants who affirmed the question. A higher percentage of excluded participants (5.8% of *n* = 103) compared to included participants (2.5% of *n* = 557) affirmed the question but the difference was not significant (Fisher's exact test, *p* = 0.108).

The first confirmed infection with the novel coronavirus (SARS-CoV-2) in Germany occurred on January 27, 2020. The course of the COVID-19 pandemic in Germany since then has been divided into several phases (Schilling et al., 2021). The first phase began in early March 2020, when the first wave started which led to a first nationwide shutdown starting on March 23. The end of the first shutdown was also the end of the first phase and was marked by the gradual reopening of the gastronomy, around May 11. The second phase of the pandemic entailed the summer months and early autumn, from the middle of May until nearly the end of September 2020. In these months, infection rates were much lower, and restrictions were more liberal, although never completely lifted. According to this scheme, we divided our participants into two groups. Participants from the beginning of March 2020 onward were assigned to the during-pandemic group, *n* = 217. Participants before March 2020 were assigned to the pre-pandemic group, *n* = 340. Our pre-pandemic group consisted of *n* = 232 participants who answered the questionnaire before the first SARS-CoV-2 infection was reported in Germany and *n* = 108 participants between January 27 and the end of February, when infections were already reported but the first wave had not yet started. Our during-pandemic group consisted of *n* = 100 participants during the first phase, including the first lockdown, and *n* = 117 participants in the summer months of the second phase, between May 12 and the end of August 2020. Sociodemographic information is reported in Table 1. Except for the group comparisons examining potential effects of the COVID-19 pandemic on mental health, all analyses were performed in the full sample. Since only two participants (0.4%) identified as diverse, we excluded this category from all analyses involving gender.

**Table 1:** Sociodemographic information.

**Variable**	**Full sample (*n* = 557)**	**Pre-pandemic (*n* = 340)**	**During-pandemic (*n* = 217)**	***U*/χ^2^(*df*)**	***p*-value**
Age (years): *M* (*SD*)	24.4 (3.4)	24.4 (3.5)	24.3 (3.3)	*U* = 37,328	0.812
Gender: *n* (%)				$\chi_{\left(1\right)}^{2}$ = 9.07	**0.003**
Female	402 (72.2)	261 (76.8)	141 (65.0)		
Male	153 (27.5)	78 (22.9)	75 (34.6)		
Diverse	2 (0.4)	1 (0.3)	1 (0.5)		
Education: *n* (%)				*U* = 33,280	0.027
No degree	3 (0.5)	2 (0.6)	1 (0.5)		
In school	7 (1.3)	4 (1.2)	3 (1.4)		
Secondary	24 (4.3)	13 (3.8)	11 (5.1)		
Advanced	302 (54.2)	201 (59.1)	101 (46.5)		
University degree	221 (39.7)	120 (35.3)	101 (46.5)		
Occupational status:^*^n (%)					
Full-time	116 (20.8)	68 (20.0)	48 (22.1)	$\chi_{\left(1\right)}^{2}$ = 0.36	0.548
Part-time	196 (35.2)	115 (33.8)	81 (37.3)	$\chi_{\left(1\right)}^{2}$ = 0.71	0.399
Studying or training	340 (61.0)	221 (65.0)	119 (54.8)	$\chi_{\left(1\right)}^{2}$ = 5.75	0.016
In school	16 (2.9)	8 (2.4)	8 (3.7)	$\chi_{\left(1\right)}^{2}$ = 0.84	0.358
Not working	48 (8.6)	23 (6.8)	25 (11.5)	$\chi_{\left(1\right)}^{2}$ = 3.80	0.051

*The gender group comparison only included the categories female and male. P-values still significant after Bonferroni-Holm correction are depicted in bold. M, mean; SD, standard deviation; df, degrees of freedom*.

* *Multiple answers possible*.

### Measures

A package of questionnaires with a mean time expenditure of 35.82 (*SD* = 16.77) minutes was administered. Here, we only describe scales included in the present analysis.

We applied the original 17-item version of the DARS (Rizvi et al., 2015) in a German translation by selecting only those items from an extended 26-item version which has been translated to German at the Ruhr-Universität Bochum, Germany, in conjunction with the scale authors (Blackwell et al., 2018). The DARS is rated on a five-point Likert scale from 0 (not at all) to 4 (very much), higher values indicating less anhedonia. All items are summed up to a total score in the range of 0 to 68. For each of the four hedonic domains, hobbies (four items, sum score 0–16), food/drink (four items, sum score 0–16), social activities (four items, sum score 0–16) and sensory experiences (5 items, sum score 0–20), participants are asked to provide two or three of their own favorite examples.

The 14 items of the SHAPS (Snaith et al., 1995; Franz et al., 1998) are rated on four categories: definitely agree (1), agree (2), disagree (3), definitely disagree (4). The original coding dichotomized the ratings into 0 (definitely agree and agree) and 1 (definitely disagree and disagree). Here, we employed a 1 to 4 ordinal scoring instead, which has also been established in the literature and can yield more dispersion in the data (Franken et al., 2007; Liu et al., 2012). The total sum score thus ranges from 14 to 56, higher values reflecting increased levels of anhedonia.

The items of CPAS and CSAS (Chapman et al., 1976, 1980; Bailer et al., 2004) have a true-false format and are encoded as 1 if answered in the direction of anhedonia. We applied a slightly shortened 50-item German version of the CPAS (Scherbarth-Roschmann and Hautzinger, 1991).

The TEPS (Gard et al., 2006; Simon et al., 2018), 18 items, and the ACIPS (German translation by D.C. Gooding and K. Kirst in 2015, personal communication; Gooding and Pflum, 2014), 17 items, are rated on a six-point Likert scale from 1 (very false for me) to 6 (very true for me), where higher ratings reflect more trait hedonic capacity.

The Behavioral Inhibition System and Behavioral Activation System (BIS/BAS) scales (Carver and White, 1994; Strobel et al., 2001) measure trait dispositions in BIS and BAS sensitivity. BIS (seven items) examines sensitivity to punishment, whereas BAS (13 items) is divided into three subscales, namely drive, fun seeking and reward responsiveness. Items are rated on a four-point Likert scale from 1 (fully disagree) to 4 (fully agree), higher values indicating more sensitivity.

Furthermore, we applied the Positive and Negative Affect Schedule (PANAS; Watson et al., 1988; Breyer and Bluemke, 2016) to measure affect on a trait level. The PANAS comprises two 10-item scales targeting positive affect (PA) and negative affect (NA). The items are rated on a five-point Likert scale from 1 (not at all) to 5 (extremely).

Depressive symptoms during the last week were assessed with a 15-item German adaptation of the Center for Epidemiologic Studies-Depression Scale (CES-D; Radloff, 1977; Hautzinger and Bailer, 1993). The items were rated on a four-point Likert scale from 0 (rarely or none of the time) to 3 (most or all of the time) and summed up to a total score in the range of 0 to 45, higher values reflecting more depressive symptoms.

### Statistical Analysis

Data cleaning was performed in Python 3.7, statistical analyses in R 4.0.2 (R Core Team, 2020). A significance level of α = 0.05 was applied for all analyses. To account for multiple testing, we report *p*-values adjusted by the modified Bonferroni procedure of Holm (1979). The Bonferroni-Holm correction is more powerful in detecting true effects than the standard Bonferroni procedure while still limiting the type 1 error to α (Aickin and Gensler, 1996; Olejnik et al., 1997). Instead of dividing the α level of every test by the number of all tests, the Bonferroni-Holm procedure orders the test results from the smallest to the highest *p*-value and adjusts the α or *p*-values sequentially. Here, the smallest *p*-value is multiplied with the number of all tests *n*, the second smallest with *n*-1 tests and so forth. The procedure is stopped when the first test becomes insignificant and all following tests are designated as insignificant (Aickin and Gensler, 1996). In our analyses, a statistical test is deemed significant after multiple testing correction if the Bonferroni-Holm corrected *p*-value is equal to or smaller than 0.05.

We performed CFA to assess the DARS factor structure using the lavaan package, version 0.6–6 (Rosseel, 2012) and robust maximum likelihood (MLR) as method of estimation. Although not developed for ordinal data, MLR was found to perform well with non-normal ordinal data of sufficient sample size and at least five categories (Byrne, 2012; Bandalos, 2014; Li, 2016), as in our case. MLR provides robust versions of fit indices and standard errors. We examined the previously reported correlated four-factor model, differentiating four hedonic domains. In order to further analyze the multidimensionality of the DARS, we additionally computed an orthogonal bifactor model with one general hedonic capacity factor and four domain group factors (Reise, 2012). Each item was specified to load onto one group factor as well as the general factor. For both models, covariances of error terms were restricted to zero. In order to address the question whether the DARS can differentiate between hedonic components as well as domains, we further analyzed a correlated four-factor model mapping onto the components interest, motivation, effort and consummatory pleasure and an eight-factor model including components and domains. We evaluated model fit based on the robust versions of six fit indices: comparative fit index (CFI), Tucker Lewis index (TLI), root mean square error of approximation (RMSEA) with a 90% confidence interval (CI), standardized root mean square residual (SRMR), Bayesian Information Criterion (BIC) and Akaike Information Criterion (AIC). We interpreted values higher than 0.95 for CFI and TLI, and values smaller than 0.06 for RMSEA and 0.08 for SRMR as cut-off criteria for good fit (Hu and Bentler, 1999). In addition, CFI and TLI over 0.90 and RMSEA under 0.10 were deemed acceptable (Marsh et al., 2004; Hopwood and Donnellan, 2010). The χ^2^-test of absolute fit is reported but known to be overly sensitive in large samples (Bentler and Bonett, 1980). Lower AIC and BIC values indicate a better fit when comparing different models.

As measures of internal consistency, we report Cronbach's α, as well as McDonald's ω coefficients which were based on the bifactor model. Omega coefficients take the multidimensionality of the scale into account and allow to estimate the variance accounted for by all factors (omega total ω_t_) as well as the variance specifically explained by the general factor (omega hierarchical ω_h_) (Revelle and Condon, 2019). High hierarchical values (ω_h_ > 0.8) would indicate unidimensionality (Rodriguez et al., 2016). Omega coefficients can also be computed for each subscale. Omega total then estimates the variance accounted for by the general and the respective group factor combined. Omega hierarchical subscale (ω_hs_) estimates the variance accounted for by the group factor after controlling for the variance attributed to the general factor (Rodriguez et al., 2016).

Due to the non-normal distribution of the DARS sum scores, we utilized Spearman rank correlations to assess convergent and divergent validity.

Differences in state anhedonia and depression between the pre-pandemic and during-pandemic groups were first examined as manifest sum score variables using Mann-Whitney *U* tests and Bonferroni-Holm adjusted *p*-values. We compared DARS total, SHAPS and CES-D scores and hypothesized to find higher CES-D scores for the during-pandemic group. Since an increase in depressive symptoms during the COVID-19 pandemic has been widely reported, we performed a one-sided test for the CES-D comparison. However, as there is little evidence regarding the effects of the pandemic on state hedonic capacity to date, we used two-sided tests for the DARS and SHAPS scores. In an exploratory *post-hoc* analysis, we examined potential group differences in the DARS domain subscales. The pre- and during-pandemic groups differed on several sociodemographic variables, namely percentage of women, education level and percentage of participants currently studying or training, see Table 1 for details. In order to control for the influence of these variables on differences in mental health, we additionally performed simple and multiple robust regressions using a MM-estimator.

Since differences between both groups might also occur on the latent level, we secondly computed multigroup CFA for the DARS. We assessed whether the COVID-19 pandemic had an impact on the measurement model of the DARS and compared the latent factor means. Measurement invariance on the configural, metric and scalar level is a necessary condition for a meaningful latent mean comparison (Putnick and Bornstein, 2016). Configural invariance implies that the basic form of the model is equivalent in both groups, all items load onto the same factors. Configural invariance is assessed by the overall fit of the model. Metric invariance means that the item loadings are similarly high across both groups. This is tested by constraining the loadings to be equal and comparing model fit to the unconstrained configural model. If the item intercepts are equal across both groups, the model is scalar invariant. This is tested by constraining the intercepts to be equal across both groups and comparing model fit to the metric model. Several criteria exist to compare model fit between two models. Here, we followed two common criteria. The change in CFA (ΔCFA) should not exceed −0.01 and the χ^2^ difference test should not be significant. Latent mean group comparisons were computed by restricting the latent means in the pre-pandemic group to zero. The estimated latent means of the during-pandemic group then indicate the unstandardized differences to the first group (Putnick and Bornstein, 2016).

## Results

### Influence of Gender and Age on the DARS

Our sample, *n* = 557, was on average 24.4 years old and 72.2% female. 54.2% of our participants had an advanced education level and 61% were currently either studying or enrolled in a job training program. For more details, see Table 1. We found similar DARS total (*Mdn* = 55, *M* = 54.43, *SD* = 8.49) and subscale scores (hobbies: *Mdn* = 14, *M* = 13.67, *SD* = 2.59; food/drink: *Mdn* = 12, *M* = 12.09, *SD* = 3.13; social activities: *Mdn* = 14, *M* = 12.89, *SD* = 2.77; sensory experiences: *Mdn* = 16, *M* = 15.77, *SD* = 3.41) as reported in previous non-clinical samples (Rizvi et al., 2015). The DARS total score was slightly elevated for female (*Mdn* = 56, *M* = 54.87, *SD* = 8.34) compared to male participants (*Mdn* = 54, *M* = 53.25, *SD* = 8.84) as indicated by a Mann-Whitney *U* test (*U* = 34532, *p* = 0.025). We found no correlation to age (*r*_*s*_ = 0.00, *p* = 0.986).

### Confirmatory Factor Analysis

CFA standardized loadings and between factor correlations for both the correlated four-factor domain and the bifactor domain DARS model are reported in Table 2. As expected, the robust fit indices indicated an acceptable to good fit for the correlated four-factor model (CFI = 0.933, TLI = 0.920, RMSEA = 0.061 [90% CI = 0.053–0.069], SRMR = 0.054, AIC = 21,225.19, BIC = 21,398.09, $\chi_{\left(113\right)}^{2}$ = 306.04), thereby confirming the differentiation of four hedonic domains: hobbies, food/drink, social activities and sensory experiences. All items loaded above 0.5 on their respective factor. For the bifactor model, fit indices were slightly improved (CFI = 0.954, TLI = 0.938, RMSEA = 0.054 [90% CI = 0.045-0.062], SRMR = 0.043, AIC = 21,154.76, BIC = 21,375.22, $\chi_{\left(102\right)}^{2}$ = 242.25). Table 2 shows several substantial loadings on the general as well as on the orthogonal domain group factors, with some items loading higher on the general and some higher on their respective group factor. The four-factor component model did not successfully differentiate between the factors interest, motivation, effort and consummatory pleasure. Although the CFA converged, estimates of between factor correlations were >1, indicating an extreme misspecification with regard to the underlying data. The output (CFI = 0.574, TLI = 0.488, RMSEA = 0.154 [90% CI = 0.147–0.162], SRMR = 0.113, AIC = 22,489.64, BIC = 22,662.54, $\chi_{\left(113\right)}^{2}$ = 1362.02) should therefore be dismissed. The eight-factor CFA model, including components and domains, did not converge.

**Table 2:** CFA standardized factor loadings and between factor correlations.

**Item**		**Four-factor model**				**Bifactor model**				
**No**.	**Domain, component**	**H**	**F/D**	**Soc**.	**Sens**.	**Gen**.	**H**	**F/D**	**Soc**.	**Sens**.
1.	Hobbies, cons. pleasure	0.768				0.589	0.499			
2.	Hobbies, effort	0.601				0.448	0.395			
3.	Hobbies, interest	0.837				0.417	0.775			
4.	Hobbies, interest	0.844				0.517	0.662			
5.	Food/drinks, motivation		0.715			0.429		0.539		
6.	Food/drinks, cons. pleasure		0.628			0.535		0.381		
7.	Food/drinks, interest		0.772			0.302		0.792		
8.	Food/drinks, effort		0.576			0.206		0.561		
9.	Social activities, cons. pleasure			0.696		0.596			0.363	
10.	Social activities, interest			0.818		0.596			0.551	
11.	Social activities, motivation			0.565		0.420			0.373	
12.	Social activities, effort			0.812		0.567			0.612	
13.	Sensory experiences, motivation				0.608	0.473				0.373
14.	Sensory experiences, interest				0.629	0.342				0.552
15.	Sensory experiences, con. pleasure				0.729	0.536				0.509
16.	Sensory experiences, interest				0.727	0.427				0.612
17.	Sensory experiences, effort				0.572	0.404				0.382
Factor correlations										
Hobbies		1	0.370	0.453	0.384					
Food/drinks			1	0.356	0.376					
Social activities				1	0.584					
Sensory experiences					1					

*N = 557. The general and the domain group factors were all specified as orthogonal for the bifactor model. See Rizvi et al. (2015) for the exact item wording. H, hobbies; F/D, food/drink; Soc., social activities; Sens., sensory experiences; Gen., general factor; cons. pleasure, consummatory pleasure*.

### Internal Consistency Analysis

In line with the factor loadings of the bifactor model, the omega coefficients ω_t_ = 0.91 and ω_h_ = 0.64 corroborated the multidimensionality of the DARS. For each subscale, ω_t_ was acceptable to high – hobbies (0.85), food/drink (0.77), social activities (0.81) and sensory experiences (0.79) – and ω_hs_, indicating the variance accounted for by the respective group factor alone, was still substantial – hobbies (0.55), food/drink (0.57), social activities (0.34) and sensory experiences (0.41). Cronbach's α for the DARS total scale and its subscales were: DARS total (0.86), hobbies (0.85), food/drink (0.76), social activities (0.81) and sensory experiences (0.79).

### Convergent and Divergent Validity

Spearman rank correlations between the DARS and other anhedonia scales are reported in Table 3. We found moderate to high correlations for the DARS total score. The total score correlated significantly higher with the anticipatory compared to the consummatory subscale of the TEPS, as indicated by Steiger's test (*z* = 3.89, *p* < 0.001). The DARS social activities subscale correlated significantly higher with the CSAS compared to CPAS (*z* = 4.74, *p* < 0.001) and with the ACIPS compared to the TEPS total score (*z* = 3.10, *p* = 0.002). Table 4 depicts the correlations to other related or diverging constructs. All hypotheses of convergent and divergent validity were confirmed.

**Table 3:** Correlations between the DARS and other anhedonia scales.

**DARS**	**SHAPS**	**CPAS**	**CSAS**	**ACIPS**	**TEPS ant**	**TEPS con**
Total scale	−0.50^***^	−0.36^***^	−0.39^***^	0.46^***^	0.51^***^	0.37^***^
Hobbies	−0.44^***^	−0.25^***^	−0.22^***^	0.25^***^	0.35^***^	0.27^***^
Food/drink	−0.28^***^	−0.17^*^	−0.13^*^	0.19^**^	0.38^***^	0.20^***^
Social activities	−0.43^***^	−0.30^***^	−0.48^***^	0.47^***^	0.34^***^	0.28^***^
Sensory experiences	−0.32^***^	−0.28^***^	−0.25^***^	0.36^***^	0.36^***^	0.30^***^

*N = 557. Spearman rank correlations with Bonferroni-Holm corrected p-values are reported. DARS, Dimensional Anhedonia Rating Scale; SHAPS, Snaith-Hamilton Pleasure Scale; CPAS, Revised Chapman Physical Anhedonia Scale; CSAS, Revised Chapman Social Anhedonia Scale; ACIPS, Anticipatory and Consummatory Interpersonal Pleasure Scale; TEPS ant, Temporal Experience of Pleasure Scale anticipatory subscale; TEPS con, Temporal Experience of Pleasure Scale consummatory subscale*.

* *Adjusted p < 0.05*.

** *Adjusted p < 0.001*.

*** *Adjusted p < 0.0001*.

**Table 4:** Correlations between the DARS and other related or diverging constructs.

**DARS**	**BAS reward**	**BAS drive**	**BAS fun**	**BIS**	**PA**	**NA**	**CES-D**
Total scale	0.36^***^	0.31^***^	0.25^***^	0.05	0.41^***^	−0.15^*^	−0.29^***^
Hobbies	0.21^***^	0.22^***^	0.17^*^	0.02	0.28^***^	−0.14^*^	−0.29^***^
Food/drink	0.21^***^	0.19^**^	0.16^*^	0.06	0.18^**^	−0.08	−0.17^*^
Social activities	0.31^***^	0.26^***^	0.24^***^	0.00	0.40^***^	−0.19^**^	−0.30^***^
Sensory experiences	0.32^***^	0.24^***^	0.16^*^	0.06	0.28^***^	−0.03	−0.08

*N = 557. Spearman rank correlations with Bonferroni-Holm corrected p-values are reported. DARS, Dimensional Anhedonia Rating Scale; BAS reward, Behavioral Activation System reward responsiveness subscale; BAS drive, Behavioral Activation System drive subscale; BAS fun, Behavioral Activation System fun seeking subscale; BIS, Behavioral Inhibition System Scale; PA, Positive Affect; NA, Negative Affect; CES-D, Center for Epidemiologic Studies-Depression Scale*.

* *Adjusted p < 0.05*.

** *Adjusted p < 0.001*.

*** *Adjusted p < 0.0001*.

### Group Comparisons

#### Manifest Differences in Depression and State Anhedonia

Detailed results of the sum score group comparisons are reported in Table 5. We found no indication of changes in state anhedonia due to the pandemic. Neither SHAPS nor DARS total scores differed significantly between pre- and during-pandemic participants. In addition, exploratory analysis revealed no differences on any DARS domain subscale (all *p* > 0.2). In line with our hypothesis, depression severity, as measured by the CES-D, was slightly but significantly elevated in the during-pandemic as compared to the pre-pandemic group. In the simple regression, pandemic-group significantly predicted CES-D scores (*n* = 557, β = 1.59, *p* = 0.041). Importantly, the effect of pandemic-group on CES-D was not weaker in the multiple regression including the three sociodemographic variables percentage of women, education level and percentage of participants currently studying or training (*n* = 555, β = 1.84, *p* = 0.022). None of the sociodemographic variables reached significance (all *p* > 0.05).

**Table 5:** State anhedonia and depression severity sum score group comparisons.

**Measure**	**Pre-pandemic (*****n*** **=** **340)**		**During-pandemic (*****n*** **=** **217)**		***U***	***p* (adjusted *p*)**
	***M* (*SD*)**	***Mdn***	***M* (*SD*)**	***Mdn***		
DARS total	54.20 (8.69)	55	54.78 (8.18)	56	35,852	0.575
Hobbies	13.61 (2.55)	14	13.77 (2.65)	15	34,799	0.248
Food/drink	12.03 (3.13)	12	12.19 (3.14)	13	35,488	0.446
Social	12.75 (2.89)	13	13.11 (2.56)	14	34,560	0.204
Sensory	15.81 (3.33)	16	15.71 (3.54)	16	37,021	0.943
SHAPS	22.46 (5.63)	22	22.88 (5.82)	22	35,466	0.441
CES-D	12.94 (8.71)	11.5	14.42 (8.70)	13	32,718	0.012 (0.036)^*^

*The Bonferroni-Holm correction did not include the DARS subscales since these comparisons were deemed exploratory. DARS total, Dimensional Anhedonia Rating Scale total scale; SHAPS, Snaith-Hamilton Pleasure Scale; CES-D, Center for Epidemiologic Studies-Depression Scale; M, mean; SD, standard deviation; Mdn, median*.

* *Significant after Bonferroni-Holm correction*.

#### DARS Multigroup CFA

Measurement invariance on a configural, metric and scalar level was established. The configural models for the correlated four-factor version (CFI = 0.931, TLI = 0.917, RMSEA = 0.062 [90% CI = 0.053–0.071], SRMR = 0.057, AIC = 21,287.78, BIC = 21,780.55, $\chi_{\left(226\right)}^{2}$ = 433.55) and the bifactor version (CFI = 0.947, TLI = 0.929, RMSEA = 0.058 [90% CI = 0.048–0.067], SRMR = 0.049, AIC = 21,234.67, BIC = 21,822.54, $\chi_{\left(204\right)}^{2}$ = 375.48) showed acceptable to good fit. Model fit did not significantly worsen by adding constraints on item loadings and intercepts. Details on the metric and scalar model comparisons are reported in Table 6. Note that only for the model comparisons we used the maximum likelihood estimator and normal, not robust, fit indices. For this reason, CFI and χ^2^ of the configural models described above differ from the CFI and χ^2^ reported in Table 6. We did this to avoid any effects of potentially differing scaling factors on the model comparisons. Table 7 depicts the results of the latent mean group comparisons of both the four-factor and the bifactor DARS model. No significant group differences were found.

**Table 6:** Tests for measurement invariance of the correlated four-factor and the bifactor DARS model.

**Model**	**χ^2^ (*df*)**	**CFI**	**Model comparison**	**Δ*χ*^2^ (Δ*df*)**	***p***	**ΔCFI**	**Decision**
Four-factor model
Configural invariance	508.70 (226)	0.922	–	–	–	–	–
Metric invariance	526.81 (239)	0.920	Configural	18.12 (13)	0.153	−0.002	Accept
Scalar invariance	537.11 (252)	0.921	Metric	10.30 (13)	0.669	0.001	Accept
Bifactor model
Configural invariance	411.59 (204)	0.942	–	–	–	–	–
Metric invariance	432.89 (233)	0.945	Configural	21.30 (29)	0.848	0.003	Accept
Scalar invariance	442.28 (245)	0.945	Metric	9.39 (12)	0.670	0	Accept

*N = 557. Measurement invariance across pre- and during-pandemic groups. For the purpose of model comparison, maximum likelihood estimation was used. M, mean; df, degrees of freedom; CFI, comparative fit index*.

**Table 7:** DARS latent means group comparisons.

**Latent factor**	**Pre-pandemic**	**During-pandemic**		***z***	***p***
	***M***	***M***	**95% CI**		
Four-factor model
Hobbies	0	0.028	[−0.081, 0.137]	0.505	0.613
Food/drink	0	0.038	[−0.114, 0.190]	0.489	0.625
Social	0	0.077	[−0.013, 0.167]	1.677	0.094
Sensory	0	−0.026	[−0.128, 0.075]	−0.503	0.615
Bifactor model
General factor	0	0.036	[−0.107, 0.178]	0.492	0.623
Hobbies	0	−0.009	[−0.120, 0.101]	−0.164	0.870
Food/drink	0	0.007	[−0.138, 0.152]	0.096	0.923
Social	0	0.040	[−0.044, 0.125]	0.932	0.351
Sensory	0	−0.045	[−0.147, 0.057]	−0.861	0.389

*Pre-pandemic group: n = 340; during-pandemic group: n = 217. The latent factor means of the pre-pandemic group were set to 0. The means of the during-pandemic group indicate the unstandardized differences between both groups. The four-factor model entails correlated factors, the factors of the bifactor model were specified as orthogonal. M, mean; CI, confidence interval*.

## Discussion

Overall, our analyses resulted in good psychometric properties for the 17-item German DARS. In a sample of 557 young, mentally healthy adults, we confirmed the differentiation of four factors mapping onto the hedonic domains. All six fit indices indicated acceptable or good fit for the correlated four-factor domain model and each item loaded substantially on the assigned factor. Since the DARS measures the same constructs, namely interest/desire, motivation, effort and consummatory pleasure, in different domains, it is reasonable to assume a general factor in addition to the specific domain factors. When a multiple correlated factor model fits the data well, it is not surprising to find a similarly well-fitting bifactor model since the correlation between the factors already indicates shared variance. But computing a bifactor model and the corresponding ω coefficients has specific advantages in assessing multidimensional scales. Bifactor models and omega coefficients can estimate how much variance is explained by each factor (Revelle and Condon, 2019). This is especially relevant when deciding on the use of specific subscales. If the items of a subscale load much higher on the general factor than on their specific group factor, it would be unwarranted to assume that the subscale score reflects a specific subconstruct or domain rather than the overall factor (Rodriguez et al., 2016). In addition, bifactor models may be useful in structural equation modeling. It is easy to integrate the general factor into a structural model and also possible to evaluate whether a group factor can predict an external variable over and above the general factor (Chen et al., 2006). Which model will be most appropriate, e.g., in future studies, therefore will depend on the purpose of the specific study. In our study, the bifactor model provides a more detailed analysis of the sources of variance in the DARS. Although the general factor explained most variance overall, we found ω_h_ to be substantially lower than ω_t_, indicating that relevant portions of variance were explained by the specific domain factors. Except for the social activities domain, more variance was explained by the domain group factors than by the general factor on the subscale level. This is also reflected in the standardized factor loadings of the bifactor model where several items loaded higher onto their respective group domain factor than onto the general factor. Among the domain subscales, food/drink showed the highest domain-specific variance and social activities the lowest. Cronbach's α suggested good reliability for the DARS total scale and acceptable to good reliability for its subscales. Our reliability results were slightly lower than the internal consistency reported by Rizvi et al. (2015) and Arrua-Duarte et al. (2019).

As expected, the DARS correlated moderately to highly with other measures of anhedonia or hedonic capacity, thereby corroborating its convergent validity. Specifically, the high correlation between the DARS total score and the SHAPS, *r*_*s*_ = −0.50, resembles the findings of Rizvi et al. (2015) in a healthy sample and Arrua-Duarte et al. (2019) in a diverse clinical sample. The higher correlation of the DARS total score to the TEPS anticipatory compared to the consummatory subscale could be explained by the DARS's inclusion of interest/desire, motivation and effort as three components besides consummatory pleasure. Possibly, anticipation, as measured by the TEPS, connects stronger to these aspects than consummatory pleasure does. Convergent validity also arises from the patterns of correlations between the DARS subscales and other anhedonia measures, especially from the stronger correlations of the subscale social activities to social compared to physical trait anhedonia scales. Moderate correlations of the DARS total score to related constructs, i.e., PA, BAS reward sensitivity and drive, also confirmed its convergent validity. Only the correlation to BAS fun was weak, a pattern also reported by Rizvi et al. (2015) in a community sample. The lack of correlation between the DARS total scale and punishment sensitivity, as measured by the BIS, as well as the only weak correlation to the NA scale of the PANAS indicate good divergent validity. In accordance with our hypothesis, we found a weak to moderate correlation to the CES-D, weaker than Rizvi et al. (2015) reported. This, however, might have been influenced by the shortened CES-D version we applied to assess depression severity.

The DARS contributes to anhedonia research by providing a valid and reliable measure which integrates different components as well as domains of pleasure. So far, its application in German samples can be recommended in contexts where a comprehensive assessment of state anhedonia is aimed for. Importantly, we only examined its validity in young, mentally healthy adults aged 18 to 30. However, since the DARS asks the participants to provide their own examples, it is unlikely to find strong age biases (Sullivan-Toole et al., 2019). Of note, the factor structure of the 17-item DARS only allows to differentiate between different domains, not between the different reward components. The failure to differentiate between the anhedonic components interest, motivation, effort and consummatory pleasure is in line with the results of the initial development (Rizvi et al., 2015) and the Spanish validation study of the DARS (Arrua-Duarte et al., 2019). A recent study on the newly developed Positive Valence Systems Scale (PVSS; Khazanov et al., 2020), comprising six constructs of the Research Domain Criteria's Positive Valence Systems domain (e.g., reward anticipation, reward valuation, reward satiation) and seven reward types (e.g., food, hobbies, goals), similarly only found factors reflecting the reward types, not the constructs. In general, hedonic domains or reward types seem to have a stronger impact on reward-related self-report than hedonic subprocesses do. The most successful attempts to distinguish components or constructs are therefore found only within one domain, for instance anticipatory and consummatory physical pleasure in case of the TEPS (Gard et al., 2006).

In the DARS, the participants themselves provide two or three of their favorite examples for each domain. An assessment of these examples yielded similar results as reported previously (Rizvi et al., 2015; Arrua-Duarte et al., 2019). As described by Rizvi et al. (2015), examples for hobbies and social activities overlapped in categories such as arts and crafts, lifestyle/culture, leisure/games, fitness/wellness/sports, multimedia/technology, education/training and food/cuisine. However, in the social domain, the social character of the activities was emphasized. In addition, we found a category of examples drawing pleasure from interpersonal interaction, relatedness and intimacy *per se*.

The DARS differs in important aspects from other established and recently developed state anhedonia scales. In contrast to the SHAPS, it is not exclusively targeted to consummatory pleasure. In contrast to the Motivation and Pleasure Scale–Self-Report (MAP-SR; Llerena et al., 2013), a self-report measure of negative symptoms created based on the Clinical Assessment Interview for Negative Symptoms (Forbes et al., 2010), the DARS is not focused on schizophrenia. Clinically, the DARS is most frequently used in depression research (Rizvi et al., 2015; Dhami et al., 2019, 2020; Bibi et al., 2020; Dunlop et al., 2020). However, due to its comprehensive design it can be applied in a wide range of contexts, in participants from healthy and diverse clinical populations. The Specific Loss of Interest and Pleasure Scale (SLIPS; Winer et al., 2014) differs from all scales described above in assessing recent changes in anhedonia, mainly in the social domain regarding consummatory pleasure and interest (Winer et al., 2014).

We further assessed the effect of the COVID-19 pandemic on depression severity and state anhedonia by dividing our sample into a pre- and a during-pandemic group. In line with our hypothesis and many previous reports on the detrimental impact on mental health (Henssler et al., 2020; O'Connor et al., 2020; Pappa et al., 2020; Bueno-Notivol et al., 2021; Cénat et al., 2021), we found slightly but significantly higher depression scores in the during-pandemic group. Importantly, this effect was not diminished when controlling for sociodemographic differences between groups. We found no group differences in state anhedonia, as measured by DARS and SHAPS sum scores. Moccia et al. (2021) reported that the SHAPS significantly predicted depression severity in a cross-sectional survey study during the early Italian lockdown. However, they did not include pre-pandemic data on the SHAPS. Our results fit a recent meta-analysis of longitudinal studies analyzing the effects of lockdown on mental health (Prati and Mancini, 2021). Prati and Mancini (2021) found a small significant effect on mental health symptoms, including depression, but the effects on positive psychological functioning, i.e., satisfaction with life, positive affect, well-being and quality of life, did not reach significance.

Multigroup CFA also gave no indication of a pandemic effect on the DARS factor model or the latent factor means. Interestingly, the measurement model of the DARS stayed invariant even across detrimental changes to the social environment such as a nationwide lockdown and distancing policies. This speaks to the robustness of the factor model and the high generalizability across different social environments. Possibly, the hypothetical phrasing of the items, such as “I would enjoy these activities,” prevented the severe limitations on pursuing certain leisure activities from affecting the ratings. This also holds true for the SHAPS. Of course, the DARS is special in asking the participants themselves to provide their own favorite examples. The measurement invariance might therefore also be an effect of allowing variance in the examples. Some people might have reported other activities and experiences than they would have under normal circumstances. For the social activities and hobbies domains, we compared the frequencies of the categories arts and crafts, lifestyle/culture, leisure/games, fitness/wellness/sports, multimedia/technology, education/training, food/cuisine, home/garden and intimacy/shared time before and during the pandemic but found no significant differences. Possibly, these categories were too broad to detect relevant distinctions. No differences stood out when inspecting the individual examples in both groups. The during-pandemic group entailed examples which might not have been possible to pursue at the time, such as “going dancing” or “going to a concert.” However, answering this question definitively would necessitate an in-depth qualitative analysis which is beyond the scope of this paper.

Among the strengths of our study are the sample size, which is sufficiently large to reliably perform CFA, as well as the inclusion of several established and recently developed anhedonia scales. This enabled us to examine the convergent validity of the DARS more closely, related to state and trait measures of anhedonia in the social and physical domain. Regarding the pandemic group comparisons, the recency of our pre-pandemic group could be a potential benefit as it offers a more accurate estimate than comparisons to data acquired years before can.

Nevertheless, several limitations must be noted. Our sample is non-representative, predominantly female and highly educated. Due to our inclusion criteria, we only assessed young, mentally healthy adults between 18 and 30 years, thereby limiting the generalizability of our findings. Our study only included self-report measures and only assessed the effect of the COVID-19 pandemic cross-sectionally. We collected no information regarding specific stressors related to the COVID-19 pandemic and can, therefore, make no claim to the cause of the increased level of depressive symptoms in the during-pandemic group. Due to the dynamic nature of the COVID-19 pandemic, constant changes in infection rates and government-ordered mitigation strategies, the circumstances at the time point of participation may have varied strongly within the during-pandemic group.

In conclusion, the 17-item DARS in German translation seems to offer a reliable and valid alternative to the SHAPS when a more comprehensive measure of state anhedonia is needed. The correlated four-factor structure, mapping onto the hedonic domains hobbies, food/drink, social activities and sensory experiences, was corroborated. Bifactor analyses substantiated the multidimensionality of the DARS. Moreover, measurement invariance across pre- and during-pandemic groups was established. The DARS satisfied all hypotheses of convergent and divergent validity and showed acceptable to high internal consistency on all subscales. Future studies in other age groups and in diverse clinical settings should further examine the utility of the German DARS. Our results on the effects of the COVID-19 pandemic are in line with many studies all over the world reporting a deterioration of mental health and an increase in depressive symptoms. Our study also adds to the growing literature of positive functioning during the COVID-19 pandemic. In our sample, we found no indication that state hedonic capacity was affected.

## Data Availability Statement

The datasets presented in this article are not readily available because of ethical restrictions. Requests to access the datasets should be directed to Sarah A. Wellan, sarah.wellan@charite.de.

## Ethics Statement

The studies involving human participants were reviewed and approved by the ethics committee of the Charité – Universitätsmedizin Berlin. The patients/participants provided their electronic informed consent to participate in this study.

## Author Contributions

SW, AD, and HW contributed to conception and design of the study. SW and AD conducted the study and performed the statistical analysis. SW wrote the first draft of the manuscript. All authors contributed to manuscript revision, read, and approved the submitted version.

## Conflict of Interest

The authors declare that the research was conducted in the absence of any commercial or financial relationships that could be construed as a potential conflict of interest.
